# Developments in the Research Base on Reducing Exposure to Second-Hand Smoke

**DOI:** 10.3390/ijerph15091873

**Published:** 2018-08-30

**Authors:** Olivia Wynne, Billie Bonevski

**Affiliations:** School of Medicine & Public Health, University of Newcastle, University Drive Callaghan, Newcastle, NSW 2308, Australia; billie.bonevski@newcastle.edu.au

There is no safe level of second-hand smoke (SHS; or passive smoke and environmental tobacco smoke) from tobacco, and it poses a serious risk to those exposed to it [1]. The health risks of SHS are well-described and substantial [1], with children at particular risk [2]. SHS affects not only smokers, but non-smokers as well. SHS as a known human carcinogen [3]; in addition to cancer, exposure to SHS has been associated with respiratory disease, cardiovascular disease, and major bacterial infections [4]. Children exposed to SHS are more likely to suffer asthma, decreased lung function, and middle ear disease [5]. Like childhood, the in utero period is especially sensitive to the detrimental effects of tobacco exposure. In addition to later life effects, smoking while pregnant can lead to poor birth outcomes, such as preterm delivery [6]. Globally, 40% of children, 35% of female non-smokers, and 33% of male non-smokers are exposed to SHS, resulting in over 600,000 deaths related to diseases caused by SHS exposure, such as ischaemic heart disease, asthma, and lung cancer [7]. SHS contributes to 1% of worldwide mortality, with the majority of the deaths in women (47%) and children (28%) [7]. Indeed, being female is a major risk factor for SHS exposure [8].

While major strides have been made in tobacco control worldwide, further steps are needed to reduce SHS exposure. Smoke-free legislation has reduced the number of places that people can smoke in many countries, however 93% of the world’s population is living in areas without 100% smoke-free policies in place [9]. Smoking bans in public places, such as bars, restaurants, and education campuses, have been shown to reduce SHS exposure for workers and patrons [10]. However, such bans are often partial, meaning that the venue is not totally smoke-free, and has designated areas where people can smoke on site. Partial bans have been shown to be poorly enforced, with evidence of smoking and SHS exposure in the designated non-smoking locations [11]. Also, private houses are out of the reach of smoke-free legislation, meaning there are significant gaps in smoke-free enforcement. Adults and children living in multi-unit housing can be at particular risk, due to SHS migrating through air ducts, open windows, balcony doors, and stairwells [12]. People of lower socioeconomic status are more likely to smoke [13], and more likely to live in multi-unit housing [14,15] potentially leading to a “double-hit” effect of SHS exposure. 

In this special issue, we aimed to explore the latest developments in methods for minimising SHS exposure. Lepore and colleagues [16] used a randomised control trial to evaluate a multi-level intervention aimed at reducing child tobacco smoke exposure in a low-income population. The intervention group received usual care which followed clinical practice guidelines for tobacco intervention (“Ask, Advise, and Refer” [AAR]), and also received individual telephone smoking cessation counselling. The control group received standard AAR with nutritional education. Both groups saw a reduction in child SHS exposure as measured by cotinine levels, and both groups were likely to adopt protective behaviours, such as implementing smoking restriction in the car and home. The results suggest the lower-cost standard practice may be sufficiently potent to reduce child SHS exposure. Importantly, the intervention resulted in parents being 2.47 times more likely to quit smoking and remain abstinent at the 12 month follow-up time period, compared to parents in the control group. SHS exposure in children has been shown to have negative health effects [5], and low-income populations are more likely to smoke [13]. Therefore, interventions targeting low-income parents have the potential to greatly reduce SHS exposure in children, and thereby reduce the detrimental developmental and health effects, and in so doing, reduce the overall health burden caused by SHS. 

Children born to mothers who smoke are more likely to suffer a range of diseases, e.g., [17,18]. Less studied is the effect of SHS exposure during pregnancy. Pregnant women are regularly asked about their cigarette smoking, however, they are not routinely asked about other forms of tobacco exposure. An Australian study by Gould et al. [19] surveyed general practitioners and obstetricians about whether they asked pregnant women about their use of e-cigarettes, cannabis use, tobacco other than cigarettes, and exposure to SHS. They found that while 95% of general practitioners and obstetricians ask pregnant women about cigarette smoking, they less frequently ask about other types of smoking (e.g., cannabis), nicotine exposure (e.g., electronic cigarette), or SHS exposure. Current guidelines specify that clinicians ask about smoking in pregnancy, however, do not discuss cannabis, chewing tobacco, or SHS exposure. Yet, exposure to all types of tobacco can have detrimental effects on health outcomes for the mother and child. Asking about such exposures would enable monitoring for adverse effects, and may be a starting point for advising women to reduce their exposures during pregnancy. 

Tobacco control measures aim to reduce tobacco use and the risk of exposure to SHS. Graphic warning labels are one such control measure, and aim to reduce tobacco use by communicating the risks associated with smoking. The WHO Framework Convention on Tobacco Control (WHO *FCTC*) [20] recommends pictorial warning labels covering at least 50% of tobacco product packaging. Graphic labels have previously been shown to change smokers’ self-reported explicit beliefs and attitudes and intentions to quit smoking [21], yet, the potential relationship between the labels and global smoking prevalence has yet to be investigated. The study by Ngo and colleagues [22], presented in this special issue, examines the association between graphic warning labels on packaging and smoking prevalence and cigarette consumption, using worldwide data from over 60 countries. They found that countries with graphic warning labels consumed significantly less cigarettes than countries without the graphic labels. The paper provides evidence of the effectiveness of graphic warning labels in reducing smoking prevalence.

Another recommendation in the WHO *FCTC* is the implementation and enforcement of smoke-free policies as a tool to reduce SHS. As such, hospitals worldwide are becoming increasingly smoke-free. The paper by McCrabb and colleagues [23] aims to assess staff perceptions of smoke-free policies, and their practices regarding smoking cessation care. McCrabb surveyed a variety of staff working in a trauma ward, including doctors, nurses, and allied health. It was found that most respondents indicated that the total smoking ban was rarely enforced, with very few staff indicating that they themselves took action to enforce the ban. Those surveyed indicated that few patients adhere to the ban, while reporting that staff who smoked adhered to the policy more. The provision of smoking cessation care varied from 74.7% of respondents reporting that they would ask patients about their level of use, to 18.1% of staff saying they would arrange a follow up for patients. Clearly, there is more work to be done in order to achieve smoke-free hospitals. McCrabb et al. recommend increasing the training of staff, an organisational structure including definitions of who is responsible for enforcement, and developing smoke-free interventions that reduce demands on staff time.

Systematic changes, such as those suggested by McCrabb, where found to be effective strategies for enforcing smoke-free policies in the review paper by Wynne et al. [11] published in this special issue. The review examines strategies used to enforce smoke-free policies across as variety of settings. The strategy with the strongest evidence of effectiveness was policy promotion/awareness, followed by penalties, system changes, and signage. Circumstances other than enforcement strategies were found to be associated with compliance to smoke-free policies, with the strength of the ban found to be the strongest influence on the success of a policy. Total bans resulted in higher smoke-free compliance than partial bans. The type of venue and smoking status of the staff also influenced the level of compliance. However, the quality assessment of the studies reviewed determined most to be of weak methodological quality. Thus, additional comprehensive, well-designed trials are needed to determine the effectiveness of strategies to enforce smoke-free policies, and thereby reduce SHS exposures.

There is still much work to be done to achieve reductions and potential elimination of SHS exposure. Nevertheless, the papers presented in this special issue provide an assessment of the current state of SHS exposure and recommendations for ways to achieve smoke-free environments. The studies address issues related to policy and practice. The papers by Lepore et al., Gould et al., and McCrabb et al., provide evidence for potential practice changes by providing cessation counselling to low-income parents and trauma ward patients, and addressing tobacco exposures in pregnant women, while the study by Ngo et al. provides support for graphic warning labels as a policy to reduce tobacco consumption. The review by Wynne et al. assesses aspects of both policy and practice in creating smoke-free environments by looking at strategies to enforce smoke-free policies. While there is still much work to be done to reduce exposure to SHS, the papers in this special issue provide solutions and a way forward for reducing exposure to tobacco smoke worldwide. 

## References

[1] U.S. Department of Health and Human Services (2006). The Health Consequences of Involuntary Exposure to Tobacco Smoke: A Report of the Surgeon General.

[2] National Health and Medical Research Council (1997). The Health Effects of Passive Smoking: A Scientific Information Paper.

[3] California Environmental Protection Agency: Air Resources Board (2005). Proposed Identification of Environmental Tobacco Smoke as a Toxic Air Contaminant.

[4] U.S. Department of Health and Human Services (2014). The Health Consequences of Smoking -50 Years of Progress: A Report of the Surgeon General.

[5] Campbell M.A., Ford C., Winstanley M.H., Scollo M.M., Winstanley M.H. (2017). Health Effects of Secondhand Smoke for Infants and Children.

[6] Ford C., Greenhalgh E.M., Winstanley M.H., Scollo M.M., Winstanley M.H. (2015). Pregnancy and Smoking.

[7] Oberg M., Jaakkola M.S., Woodward A., Peruga A., Pruss-Ustun A. (2011). Worldwide burden of disease from exposure to second-hand smoke: A retrospective analysis of data from 192 countries. Lancet.

[8] Bonevski B., Paul C., Jones A., Bisquera A., Regan T. (2014). Smoky homes: Gender, socioeconomic and housing disparities in second hand tobacco smoke (SHS) exposure in a large population-based Australian cohort. Prev. Med..

[9] Öberg M., Prüss-Üstün A., Schweizer C., Woodward A. (2010). Second-Hand Smoke: Assessing the Environmental Burden of Disease at National and Local Levels.

[10] Greenhalgh E., Scollo M., Scollo M.M., Winstanley M.H. (2018). Effectiveness of Smokefree Legislation in Reducing Exposure to Tobacco Toxins, Improving Health, and Changing Smoking Behaviours.

[11] Wynne O., Guillaumier A., Twyman L., McCrabb S., Denham A.M.J., Paul C., Baker A.L., Bonevski B. (2018). Signs, Fines and Compliance Officers: A Systematic Review of Strategies for Enforcing Smoke-Free Policy. Int. J. Environ. Res. Public Health.

[12] Kraev T.A., Adamkiewicz G., Hammond S.K., Spengler J.D. (2009). Indoor concentrations of nicotine in low-income, multi-unit housing: Associations with smoking behaviours and housing characteristics. Tobacco Control.

[13] Australian Institute of Health and Welfare (2017). National Drug Strategy Household Survey 2016: Detailed Findings.

[14] Australian Bureau of Statistics (ABS) (2017). Housing Occupancy and Costs.

[15] U.S. Department of Housing and Urban Development (2018). Resident Characteristics Report: Public Housing. https://pic.hud.gov/pic/RCRPublic/rcrmain.asp.

[16] Lepore S., Collins B., Coffman D., Winickoff J., Nair U., Moughan B., Bryant-Stephens T., Taylor D., Fleece D., Godfrey M. (2018). Kids Safe and Smokefree (KiSS) Multilevel Intervention to Reduce Child Tobacco Smoke Exposure: Long-Term Results of a Randomized Controlled Trial. Int. J. Environ. Res. Public Health.

[17] Thacher J.D., Gehring U., Gruzieva O., Standl M., Pershagen G., Bauer C.-P., Berdel D., Keller T., Koletzko S., Koppelman G.H. (2018). Maternal Smoking during Pregnancy and Early Childhood and Development of Asthma and Rhinoconjunctivitis—A MeDALL Project. Environ. Health Perspect..

[18] Chamberlain C., O’Mara-Eves A., Porter J., Coleman T., Perlen S.M., Thomas J., McKenzie J.E. (2017). Psychosocial interventions for supporting women to stop smoking in pregnancy. Cochrane Database Syst. Rev..

[19] Gould G., Zeev Y., Tywman L., Oldmeadow C., Chiu S., Clarke M., Bonevski B. (2017). Do Clinicians Ask Pregnant Women about Exposures to Tobacco and Cannabis Smoking, Second-Hand-Smoke and E-Cigarettes?. An Australian National Cross-Sectional Survey. Int. J. Environ. Res. Public Health.

[20] World Health Organization (2003). Framework Convention on Tobacco Control. http://www.who.int/fctc/cop/art%208%20guidelines_english.pdf.

[21] Macy J.T., Chassin L., Presson C.C., Yeung E. (2016). Exposure to graphic warning labels on cigarette packages: Effects on implicit and explicit attitudes towards smoking among young adults. Psychol. Health.

[22] Ngo A., Cheng K.W., Shang C., Huang J., Chaloupka F.J. (2018). Global Evidence on the Association between Cigarette Graphic Warning Labels and Cigarette Smoking Prevalence and Consumption. Int. J. Environ. Res. Public Health.

[23] McCrabb S., Baker A.L., Attia J., Balogh Z.J., Lott N., Palazzi K., Naylor J., Harris I.A., Doran C.M., George J. (2017). Hospital Smoke-Free Policy: Compliance, Enforcement, and Practices. A Staff Survey in Two Large Public Hospitals in Australia. Int. J. Environ. Res. Public Health.

